# The impact of increasing non-albicans *Candida* trends on diagnostics in immunocompromised patients

**DOI:** 10.1007/s42770-023-01163-3

**Published:** 2023-11-08

**Authors:** Rasha M. Abdel-Hamid, Hadir A. El-Mahallawy, Nesma E. Abdelfattah, Mona A. Wassef

**Affiliations:** 1 Clinical Pathology Department, National Cancer Institute, Cairo University, Cairo, Egypt; 2 Clinical & Chemical Pathology Department, Faculty of Medicine, Cairo University, Cairo, Egypt

**Keywords:** Invasive candidiasis, Non-albicans *Candida*, Multiplex semi-nested PCR, Cancer patients, Rare species. Risk factor

## Abstract

Invasive candidiasis (IC) represents a growing concern worldwide, with a considerable increase in non-albicans *Candida* (NAC) species. The study's primary goal was to determine if species identification by semi-nested PCR (sn-PCR) with primers for the five most prevalent *Candida* species is sufficient to deal with the current trends of *Candida* infections in cancer patients. Over one year, *Candida* isolates were collected from samples of patients with hematological and solid organ tumors in a single center. Species of *Candida* were identified by chromagar and multiplex sn-PCR using specific primers for *Candida albicans*, *Candida tropicalis*, *Candida glabrata*, *Candida krusei*, and the *Candida parapsilosis* complex. Most *Candida* infection episodes are caused by NAC species (70.5% of 105 isolates). Rare species (14 isolates) accounted for 13.3% of isolates and were not identified by sn-PCR using the five most common *Candida* species primers. More than half of these rare species caused candidemia in cancer patients (57.1%; *p* = 0.011). The risk factor for candidiasis was recent surgeries (*p* = 0.020) in adults and chemotherapy in pediatric patients (*p* = 0.006). Prolonged hospitalization and genitourinary tract cancer were significantly associated with invasive infections (*p* = 0.005 and 0.049, respectively). Recent surgery was a significant risk factor associated with *C. parapsilosis* and *C. glabrata* infections (*P* = 0.038 and 0.003, respectively), while *C. tropicalis was* significantly more common in patients with hematological malignancies (*P* = 0.012). Techniques with a broader identification spectrum than the major five *Candida* species are crucial for the optimal management of cancer patients.

## Introduction

Invasive *Candida* infections represent a growing concern in healthcare settings, especially among high-risk immunocompromised patients such as those who have cancer [1]. Despite *C. albicans* being the most frequent species, NAC species have increased significantly in the last two decades [2, 3]. High mortality rates and decreased antifungal susceptibility because of this shift towards NAC necessitate rapid, accurate species identification and improving our knowledge of clinical characteristics, risk factors, and outcomes associated with these pathogens to guide the clinician for optimal therapeutic interventions [2].

Significant variations in *Candida* species distribution are observed within different geographical areas. It mainly depends on the patient population, age, use of central venous catheters, broad-spectrum antibiotics, and antifungal strategies [3]. Since different *Candida* species show variable resistance patterns, rapid identification to the species level is a substantial prerequisite for convenient antimycotic therapy management, especially if antifungal susceptibility testing is not accessible [4]. Although *C. albicans* is typically susceptible to commonly used antifungals, *Candida guilliermondii* and *C. parapsilosis* may gain echinocandin resistance, *C. glabrata may gain *resistance to azoles, *Candida lusitaniae* can have diminished susceptibility to amphotericin B, and *C. krusei* has fluconazole intrinsic resistance. Moreover, *Candida auris* has recently emerged as a multiresistant healthcare-associated pathogen worldwide [5].

Many studies have found that more than 90% of invasive *Candida* infections are caused by *C. albicans*, *C. parapsilosis*, *C. tropicalis*, *C. krusei*, and *C. glabrata* species [6, 7]. PCR is a milestone in *Candida* infection diagnosis as it detects a trace amount of the microorganism's nucleic acid. Moreover, nested PCR is considered a very accurate procedure, improving the sensitivity and specificity of detecting *Candida* infections due to the low chance that any improperly amplified PCR fragment will be re-amplified in the second run [8]. Building on insights from local epidemiology is an urgent prerequisite to constructing hospital antifungal protocols. Therefore, we aimed in this study to find out whether *Candida* species identification by sn-PCR using primers for the five major *Candida* species is enough to cope with current infection patterns among cancer patients. We also assess the distribution, clinical characteristics, and patient outcomes associated with invasive infections and infections caused by different species in oncological patients.

## Methodology

### Study design and data collection

This study was conducted at Egypt's National Cancer Institute (NCI), Cairo University. Over a year, *Candida* isolates cultured from various specimens from cancer patients referred to the NCI Microbiology Laboratory were collected. Inpatients and outpatients with candidiasis who showed symptoms and signs of infection were included, with only one isolate from each patient. Demographic data, underlying diseases, clinical characteristics, and outcomes of patients were obtained retrospectively. Patients' isolates with missing data were excluded. The clinical features of patients, risk factors, and microbiological characteristics of invasive candidiasis and *Candida* infections caused by various *Candida* species were analyzed. A healthcare-associated infection (HAI) was considered when a positive culture occurred more than 48 h after admission [9]. Lymphopenia was defined as the absolute lymphocyte count (ALC) being less than 0.7 × 10^3^/μl, and neutropenia as the absolute neutrophil count (ANC) being less than 1.0 × 10^3^/μl [10].

### Candida species identification

All samples were processed under standard microbiological procedures immediately upon arrival in the laboratory. To be included, specimens must meet the following criteria: the presence of *Candida* in at least one positive blood culture with clinical symptoms and signs of infection; a pure growth of *Candida* with a significant colony count (≥ 10^5^ colony-forming units (CFU)/mL) in urine samples from symptomatic patients; and sputum specimens where 25 or more leucocytes and fewer than ten epithelial cells per low-power field (10x) [11]. All isolates were then subcultured on Sabouraud dextrose agar (SDA), wet mount, and Gram staining as primary screening for *Candida* colonies. Chromagar (CHROMAgar™ *Candida* Becton Dickinson, Germany) and multiplex sn-PCR were used for further *Candida* species identification. When a strain's species could not be determined, it was classified as an "unidentified *Candida* species."

#### Multiplex semi-nested PCR

DNA extraction was done according to the manufacturer's instructions using QIAGEN (QIAamp DNA Mini Kit). The PCR amplification was performed using the fungus-specific oligonucleotides with internal transcribed spacers 1 and 4 (ITS1 and ITS4) as outer primers (5'-TCCGTAGGTGAACCTGCGG-3' and 5'-TCCTCCGCTTATTGATATGC-3', respectively). Then the inner primers for *C. krusei*, *C. glabrata*, *C. albicans*, *C. parapsilosis* complex, and *C. tropicalis* were used. The primer sequences were published in [12] and were as follows: CKR (F- 5' ACTACACTGCGTGAGCGGAA 3') (R- 5' AAAAAGTCTAGTTCGCTCGG 3'); CGL (F- 5' TTATCACACGACTCGACACT 3'), (R- 5' CCCACATACTGATATGGCCTACAA 3'); CALB (F- 5' TTTATCAACTTGTCACACCAGA 3'), (R- 5' ATCCCGCCTTACCACTACCG 3'); CPAR (F- 5' GCCAGAGATTAAACTCAACCAA 3'), (R- 5' CCTATCCATTAGTTTATACTCCGC 3'); and CTR (F- 5' CAATCCTACCGCCAGAGGTTAT 3') (R- 5' TGGCCACTAGCAAAATAAGCGT 3'), respectively. The sizes of the amplicons were 362 bp, 423 bp, 272 bp, 297 bp, and 357 bp, respectively. The protocol described in [12] was used for DNA extraction, quantification, and amplification. The amplified PCR products were run on 2% agarose gel electrophoresis and then visualized by a UV transilluminator (Biometra).

### Statistical analysis

The qualitative data were shown as frequency and percentage, while the numerical data were shown as mean and standard deviation, or median and range, as convenient. Comparisons between qualitative variables were examined by the Chi-square or Fisher’s exact test; however, the Mann–Whitney test was used to compare continuous variables. The correlation between diagnostic techniques was examined by the Kappa test, where range values of 1% to 20% indicate minor agreement, 21% to 40% fair agreement, 41% to 60% median agreement, 61% to 80% significant agreement, and 81% to 100% nearly perfect agreement. Risk estimates were measured and expressed as an odds ratio and a 95% confidence interval (CI) to identify risk factors for different groups. The Kaplan–Meier test was used in survival analysis, and the log-rank test was used to compare survival between groups. To detect the independent risk factors for thirty-day post-infection mortality, binary logistic regression was used to analyze factors with a *p-*value less than 0.1 in univariate analysis. A *p*-value less than 0.05 was regarded as significant. The data were statistically analyzed using version 25 of IBM SPSS (SPSS Inc., Armonk, NY, USA).

## Results

### Clinical characteristics of Candida-infected cancer patients

The demographic characteristics of 105 cancer patients diagnosed with *Candida* infections during the study period are summarized in Table 1. *Candida* infections were more prevalent in the over-50 age group, followed by the 18–50 age group and children (< 18 years) (*n* = 46, 43.8%, *n* = 38, 36.2%, and *n* = 21, 20.0%, respectively). Infected children had a median age of 6 years, ranging from 1 to 17 years, whereas adults had a median age of 47 years, ranging from 19 to 80 years. Hematological malignancies were observed in 37.1% and solid organ tumors in 62.9% of all patients. Gastrointestinal tract (GIT) tumors were significantly more common in *Candida*-infected adult patients, while central nervous system (CNS) cancers were more common in children (*p* = 0.011 and *p* < 0.001, respectively). Neutropenia and lymphopenia were present in 33.3% and 42.9% of patients, respectively.

**Table 1:** Risk factors and clinical characteristics of 105 cancer patients with *Candida* infections

Characteristics	No. of cases (%)			*P*-value
	All patients (*n* = 105)	Pediatric patients (*n* = 21)	Adult patients (*n* = 84)	
Age* (median & range)	50 (1–80)	6 (1–17)	53 (19–80)	** < 0.001**
Male sex	59 (56.2)	12 (57.1)	47 (56.0)	0.922
Type of cancer				
Hematological malignancies	39 (37.1)	11 (52.4)	28 (33.3)	0.106
Gastrointestinal tract cancer	28 (26.7)	1 (4.8)	27 (32.1)	**0.011**
Genitourinary tract cancer	16 (15.2)	2 (9.5)	14 (16.7)	0.415
Central nervous system cancer	5 (4.8)	5 (23.8)	0 (0.0)	** < 0.001**
Solid cancers in other locations	17 (16.2)	2 (9.5)	15 (17.9)	0.354
Inpatients	102 (97.1)	21 (100.0)	81 (96.4)	1.000
Period of hospitalization* (median & range)	10 (3–66)	9 (3–66)	11 (3–62)	0.475
≥ 7 days hospital stay	69 (65.7)	12 (57.1)	57 (67.9)	0.355
ICU admission	56 (53.3)	10 (47.6)	46 (54.8)	0.557
Recent surgery (≤ 30 days)	38 (36.2)	3 (14.3)	35 (41.7)	**0.020**
Chemotherapy	52 (49.5)	16 (76.2)	36 (42.9)	**0.006**
Prior antibiotic use	101 (96.2)	21 (100.0)	80 (95.2)	0.581
Presence of neutropenia	35 (33.3)	10 (47.6)	25 (29.8)	0.121
Presence of lymphopenia	45 (42.9)	7 (33.3)	38 (45.2)	0.324
Laboratory findings* (median & range)				
Absolute neutrophil count (cell/μl)	5475 (0–71610)	2160 (0–19200)	5985 (0–71610)	0.228
Absolute lymphocyte count (cell/μl)	946 (0–34500)	1050 (68–5250)	870 (0–34500)	0.721
Platelet count (cell/μl)	150 (3–700)	129 (9–600)	153 (3–700)	0.532

Nominal values are shown as a number and a percentage, whereas numerical values * are shown as a median and range. Statistically significant *P*-values are shown in boldface. *ICU* intensive care unit

Most *Candida* infection episodes were HAIs (97.1%), with the median time spent in the hospital before the onset of infection being ten days. Also, the presence of *Candida* infections was linked to a longer hospital stay, admission to the intensive care unit (ICU), previous exposure to broad-spectrum antibiotics, receiving chemotherapy, and recent surgical procedures (65.7%, 53.3%, 96.2%, 49.5%, and 36.2%, respectively). Recent surgeries were a significant risk factor for candidiasis in adult patients, whereas chemotherapy was in pediatric patients (*p* = 0.020 and *p* = 0.006, respectively).

### Identification of Candida species

**Fig. 1:**
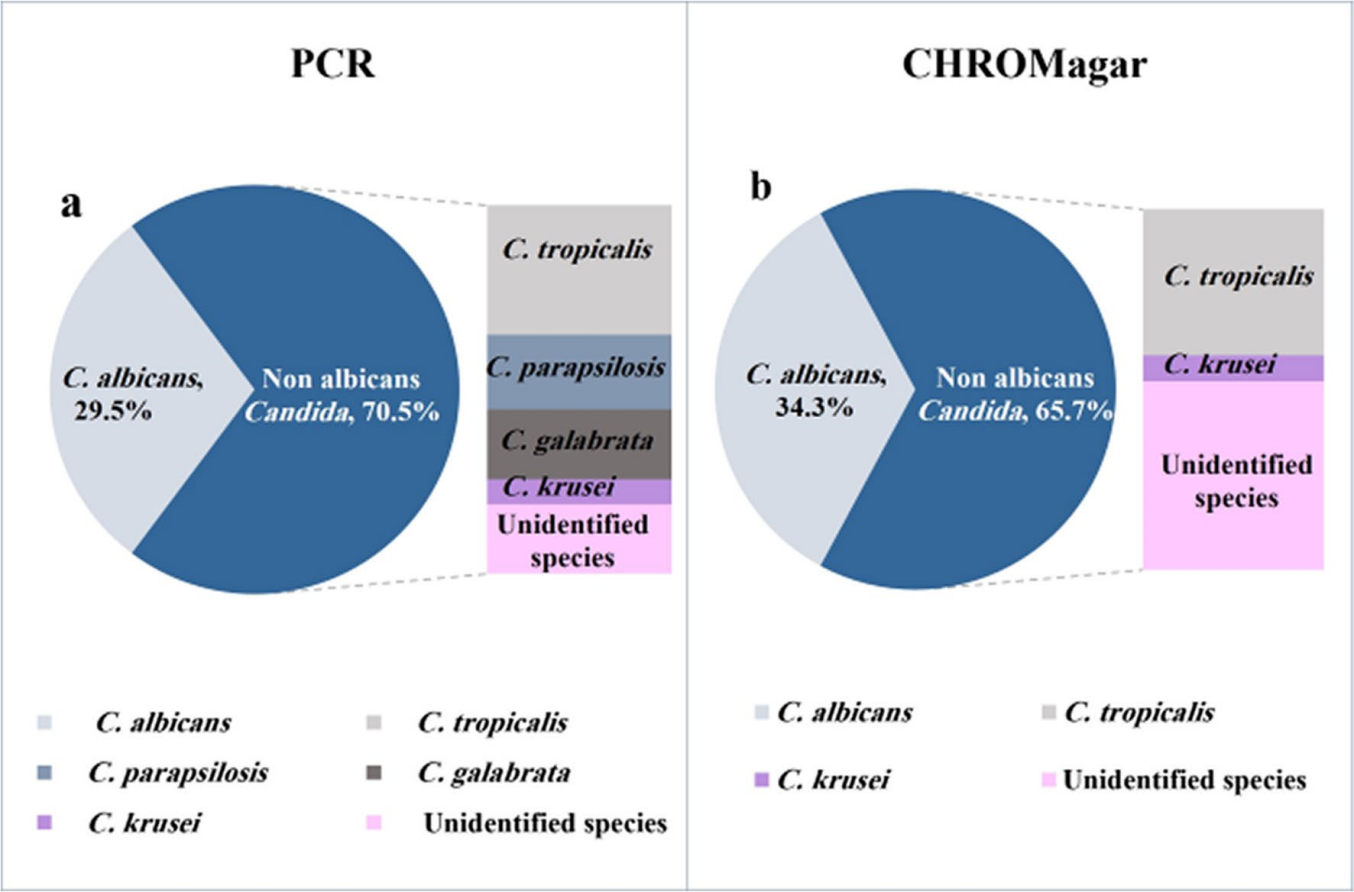
Frequency of different *Candida* species identified by multiplex semi-nested PCR (**a**) and by chromagar (**b**). non-albicans *Candida* (NAC) species represented more than 2/3 of species (n=74; 70.5% and n=69; 65.7% as identified by sn-PCR (semi-nested PCR) and chromagar, respectively). sn-PCR and chromagar were unable to identify 13.3% and 34.3% of *Candida* isolates. *C. tropicalis* and *C. krusei* were detected in 24.8% and 4.8% of all *Candida* isolates by PCR and 26.7% and 4.8% by chromagar, respectively. *C. parapsilosis* and *C. glabrata* were identified in 14.3% and 13.3% by PCR but could not be identified by chromagar

Regarding clinical sites of infection, 30 patients had candidemia, 25 had surgical site infections (SSIs), 17 had urinary tract infections (UTIs), and 33 had respiratory tract infections (RTIs) (28.6%, 23.8%, 16.2%, and 31.4%, respectively). The frequency of different *Candida* species identified by sn-PCR is shown in Fig. 1a. NAC species represented 70.5% of isolates (*n* = 74). *C. tropicalis* was the most frequently isolated NAC species, followed by *C. parapsilosis*, *C. glabrata,* and *C. krusei* (24.8%, 14.3%, 13.3%, and 4.8%, respectively). Fourteen isolates (13.3%) were not identified to the species level. Furthermore, 33 patients (31.4%) had concomitant *Candida* infections in other body sites.

Figure 1b illustrates the frequency of *Candida* species identified by chromagar. The results of chromagar showed a high degree of correspondence with those of sn-PCR, as presented in Table 2. Thus, the overall concordance of the chromagar compared to sn-PCR tests was found to be 80.7% (*p* < 0.001). There was a discrepancy between both results in 14 isolates (13.3%) (two *C. albicans*, two *C. tropicalis*, one *C. glabrata*, six *C. parapsilosis*, and three other *Candida* species as identified by sn-PCR). Besides, the exact species of 36 *Candida* isolates (34.3%) could not be determined by chromagar.

**Table 2:** Statistical agreement between chromagar and semi-nested PCR in *Candida* species identification

	sn-PCR (No of isolates)	Chromagar (No of isolates)	Agreement^a^ (%)	*P*-value
*C. albicans*			80.3	< 0.001
Positive	31	36		
Negative	74	69		
*C. tropicalis*			85.0	< 0.001
Positive	26	28		
Negative	79	77		
*C. parapsilosis*			NC	NC
Positive	15	0		
Negative	90	105		
*C. glabrata*			NC	NC
Positive	14	0		
Negative	91	105		
*C. krusei*			100.0	< 0.001
Positive	5	5		
Negative	100	100		

*sn-PCR* semi-nested PCR, *NC* non-calculable

^a^ Refers to % of agreement of chromagar with sn-PCR

### Risk factors associated with invasive candidiasis

We divided *Candida* infections into two groups based on clinical sites: invasive candidiasis (infections in sterile body sites such as the bloodstream and surgical sites) and non-invasive candidiasis (urine and respiratory tract infections). Table 3 compares the two groups' risk factors, clinical features, microbiological characteristics, and patient outcomes. Invasive *Candida* infections were found to be significantly associated with prolonged hospital stay (≥ seven days) (*p* = 0.005). Furthermore, the type of underlying malignancy was a significant variable in invasive infections. Invasive infections were significantly higher in patients with genitourinary tract cancer and lower in patients with solid cancers other than the GIT, genitourinary tract (GUT), and CNS (*p* = 0.049 and *p* = 0.038, respectively). On the other hand, age, gender, hospitalization, ICU admission, chemotherapy, recent surgery, antibiotic intake, neutropenia, lymphopenia, *Candida* species, and multifocal *Candida* infections in other body sites did not differ statistically between the two groups.

**Table 3:** Risk factors, clinical features, and microbiological characteristics of invasive candidiasis in 105 *Candida*-infected cancer patients

Risk factors (no patients)	No. of cases (%)		Odds ratio	95% CI	*P*-value
	invasive candidiasis (*n* = 55)	Non-invasive candidiasis (*n* = 50)			
Age					
< 18 years (*n* = 21)	15 (27.3)	6 (12.0)	2.750	0.973–7.773	0.051
18–50 years (*n* = 38)	20 (36.4)	18 (36.0)	1.016	0.458–2.254	0.969
> 50 years (*n* = 46)	20 (36.4)	26 (52.0)	0.527	0.242–1.152	0.107
Male sex (*n* = 59)	31 (56.4)	28 (56.0)	1.015	0.469–2.196	0.970
Type of cancer					
Hematological malignancies (*n* = 39)	19 (34.5)	20 (40.0)	0.792	0.358–1.750	0.563
Gastrointestinal tract cancer (*n* = 28)	14 (25.5)	14 (28.0)	0.878	0.369–2.087	0.768
Genitourinary tract cancer (*n* = 16)	12 (21.8)	4 (8.0)	**3.209**	**0.961–10.714**	**0.049**
Central nervous system cancer (*n* = 5)	5 (9.1)	0 (0.0)	NC	NC	0.058
Solid cancers in other locations (*n* = 17)	5 (9.1)	12 (24.0)	**0.317**	**0.103–0.976**	**0.038**
Inpatients (*n* = 102)	55 (100.0)	47 (94.0)	NC	NC	0.105
** ≥ **7 days hospital stay (*n* = 69)	43 (78.2)	26 (52.0)	**3.308**	**1.418–7.715**	**0.005**
ICU admission (*n* = 56)	30 (54.5)	26 (52.0)	1.108	0.514–2.387	0.794
Recent surgery (≤ 30 days) (*n* = 38)	20 (36.4)	18 (36.0)	1.016	0.458–2.254	0.969
Receiving chemotherapy (*n* = 52)	26 (47.3)	26 (52.0)	0.828	0.384–1.782	0.628
Prior antibiotic use (*n* = 101)	53 (96.4)	48 (96.0)	1.104	0.150–8.146	1.000
Presence of neutropenia (*n* = 35)	17 (30.9)	18 (36.0)	0.795	0.353–1.793	0.580
Presence of lymphopenia (*n* = 45)	21 (38.2)	24 (48.0)	0.669	0.308–1.455	0.310
*Candida* species					
*C. albicans* (*n* = 31)	14 (25.5)	17 (34.0)	0.663	0.285–1.540	0.338
*C. tropicalis* (*n* = 26)	12 (21.8)	14 (28.0)	0.718	0.295–1.746	0.464
*C. parapsilosis* (*n* = 15)	11 (20.0)	4 (8.0)	2.875	0.852–9.706	0.079
*C. glabrata* (*n* = 14)	7 (12.7)	7 (14.0)	0.896	0.291–2.761	0.848
Other *Candida* species^a^ (*n* = 19)	11 (20.0)	8 (16.0)	1.313	0.481–3.582	0.595
Multifocal*Candida*infections (*n* = 33)	17 (30.9)	16 (32.0)	0.951	0.417–2.169	0.904
Thirty-day mortality (*n* = 34)	18 (32.7)	16 (32.0)	1.034	0.456–2.344	0.937

*ICU* intensive care unit, *CI* confidence interval, *NC* non-calculable

^a^other species include *C. krusei* (*n* = 5) and unidentified *Candida* species (*n* = 14). Statistically significant *P*-values are in boldface

### Risk factors associated with infection by different Candida species

In the present study, *Candida* isolates were grouped into five categories according to the identified species: *C. albicans* (*n* = 31), *C. tropicalis* (*n* = 26), *C. parapsilosis* (*n* = 15), *C. glabrata* (*n* = 14), and other *Candida* species (*n* = 19), which included *C. krusei* (*n* = 5) and all unidentified species (*n* = 14). Table 4 compares groups in terms of risk factors, clinical characteristics, outcomes of patients, and associated microbiological features. *C. albicans* infections were significantly lower in lymphopenic patients (OR: 0.348, 95% CI: 0.138 to 0.877, *P* = 0.022). *C. tropicalis* was significantly more frequently detected in patients with hematological malignancies but significantly less frequently in patients who had recently undergone surgery (OR: 3.125, 95% CI: 1.253 to 7.794, *P* = 0.012; OR: 0.332, 95% CI: 0.114 to 0.970, *P* = 0.038, respectively). This species was significantly more frequently isolated from UTIs and less from RTIs (OR: 6.429, 95% CI: 2.124 to 19.456, *P* < 0.001; OR: 0.313, 95% CI: 0.098 to 0.999, *P* = 0.042, respectively).

**Table 4:** Risk factors, clinical characteristics of 105 cancer patients, and microbiological features associated with infection by different *Candida *species

Risk factors	*C. albicans (n* = *31)*		*C. tropicalis (n* = *26)*		*C. parapsilosis (n* = *15)*		*C. glabrata (n* = *14)*		*Other Candida* species ^a^ (*n* = 19)	
	OR (95% CI)	*P-value*	OR (95% CI)	*P-value*	OR (95% CI)	*P-value*	OR (95% CI)	*P-value*	OR (95% CI)	*P-value*
Age										
< 18 years	0.497 (0.152–1.619)	0.239	1.711 (0.604–4.846)	0.309	1.000 (0.255–3.922)	1.000	0.273 (0.034–2.216)	0.196	2.185 (0.715–6.670)	0.163
18–50 years	0.782 (0.322–1.900)	0.587	1.412 (0.571–3.496)	0.454	1.665 (0.552–5.021)	0.362	0.976 (0.302–3.158)	0.968	0.574 (0.189–1.740)	0.322
> 50 years	1.884 (0.807–4.397)	0.140	0.480 (0.187–1.231)	0.122	0.598 (0.189–1.889)	0.377	1.860 (0.596–5.800)	0.280	0.919 (0.336–2.510)	0.869
Male sex	0.639 (0.275–1.486)	0.297	1.664 (0.663–4.179)	0.276	**0.231 (0.068–0.784)**	**0.013**	1.476 (0.459–4.749)	0.512	2.551 (0.845–7.705)	0.089
Type of cancer										
Hematological malignancies	0.904 (0.377–2.163)	0.820	**3.125 (1.253–7.794)**	**0.012**	**0.220 (0.047–1.035)**	**0.039**	**0.107 (0.013–0.856)**	**0.013**	2.184 (0.799–5.969)	0.123
Gastrointestinal tract cancer	1.844 (0.740–4.598)	0.186	0.417 (0.130–1.340)	0.134	2.061 (0.659–6.439)	0.207	1.117 (0.320–3.897)	0.863	0.458 (0.122–1.709)	0.236
Genitourinary tract cancer	0.503 (0.133–1.906)	0.305	0.387 (0.082–1.830)	0.217	2.364 (0.647–8.636)	0.183	2.633 (0.711–9.749)	0.136	1.053 (0.268–4.131)	0.941
Central nervous system cancer	0.583 (0.063–5.439)	1.000	2.111 (0.333–13.388)	0.595	1.536 (0.160–14.760)	0.545	NC	1.000	1.139 (0.120–10.804)	1.000
Other solid tumors	0.994 (0.318–3.105)	0.991	0.606 (0.159–2.300)	0.458	0.769 (0.157–3.767)	0.746	**3.657 (1.047–12.773)**	**0.033**	0.557 (0.116–2.670)	0.459
Inpatients	0.833 (0.073–9.541)	1.000	0.649 (0.056–7.469)	1.000	NC	1.000	NC	1.000	0.429 (0.037–4.986)	0.454
** ≥ **7 days hospital stay	1.138 (0.466–2.774)	0.777	2.041 (0.736–5.655)	0.165	1.517 (0.447–5.155)	0.502	2.086 (0.543–8.017)	0.276	**0.168 (0.057–0.496)**	**0.001**
ICU admission	1.090 (0.470–2.529)	0.841	0.554 (0.226–1.358)	0.194	1.913 (0.606–6.044)	0.264	1.194 (0.384–3.719)	0.759	0.966 (0.357–2.614)	0.946
Recent surgery (≤ 30 days)	0.782 (0.322–1.900)	0.587	**0.332 (0.114–0.970)**	**0.038**	**3.155 (1.026–9.705)**	**0.038**	**5.625 (1.624–19.478)**	**0.003**	0.408 (0.125–1.333)	0.129
Receiving chemotherapy	0.780 (0.336–1.810)	0.563	2.375 (0.945–5.970)	0.062	0.457 (0.145–1.445)	0.176	**0.234 (0.061–0.894)**	**0.024**	2.611 (0.908–7.510)	0.069
Prior antibiotic use	0.403 (0.054–2.996)	0.580	NC	0.570	0.483 (0.047–4.974)	0.465	NC	1.000	NC	0.556
Presence of neutropenia	1.146 (0.474–2.768)	0.762	2.087 (0.839–5.190)	0.110	**0.118 (0.015–0.935)**	**0.018**	0.293 (0.062–1.389)	0.104	2.077 (0.755–5.710)	0.152
Presence of lymphopenia	**0.348 (0.138–0.877)**	**0.022**	2.227 (0.905–5.484)	0.078	1.197 (0.400–3.587)	0.747	1.395 (0.452–4.307)	0.562	0.963 (0.352–2.633)	0.942
Site of infection										
Blood stream infections	0.822 (0.319–2.118)	0.685	1.462 (0.566–3.780)	0.432	0.583 (0.152–2.235)	0.427	**NC**	**0.011**	**3.667 (1.309–10.272)**	**0.010**
Surgical site infections	0.695 (0.247–1.951)	0.488	0.338 (0.092–1.240)	0.090	**4.908 (1.564–15.398)**	**0.004**	**4.056 (1.262–13.037)**	**0.013**	**0.144 (0.018–1.135)**	**0.036**
Urinary tract infections	0.695 (0.208–2.328)	0.554	**6.429 (2.124–19.456)**	** < 0.001**	0.330 (0.040–2.696)	0.279	NC	0.077	0.557 (0.116–2.670)	0.459
Respiratory tract infections	1.950 (0.810–4.695)	0.133	**0.313 (0.098–0.999)**	**0.042**	0.500 (0.131–1.907)	0.303	2.500 (0.798–7.833)	0.108	1.009 (0.346–2.938)	0.988
Multifocal*Candida*infections	0.852 (0.341–2.129)	0.732	1.213 (0.474–3.104)	0.687	2.154 (0.708–6.549)	0.170	0.855 (0.247–2.957)	0.805	0.524 (0.159–1.723)	0.282
Thirty-day mortality	1.823 (0.760–4.369)	0.183	0.906 (0.348–2.357)	0.978	0.727 (0.214–2.477)	0.633	0.813 (0.236–2.808)	0.628	0.702 (0.230–2.140)	0.529

*NC* non-calculable, *ICU* intensive care unit, *CI* confidence interval

^a^other species include *C.krusei* (*n* = 5) and unidentified *Candida* species (*n* = 14). Statistically significant *P*-values are in boldface

While *C. parapsilosis* infections were significantly more common in patients who had recently undergone surgery, they were significantly less common in male patients, those with hematological malignancies, and neutropenic patients (OR: 3.155, 95% CI: 1.026 to 9.705, *P* = 0.038; OR: 0.231, 95% CI: 0.068 to 0.784, *P* = 0.013; OR: 0.220, 95% CI: 0.047 to 1.035, *P* = 0.039; OR: 0.118, 95% CI: 0.015 to 0.935, *P* = 0.018, respectively). The preceding species produced significantly more SSIs (OR: 4.908, 95% CI: 1.564 to 15.398, *P* = 0.004). However, infections by *C. glabrata* were significantly revealed in patients with solid organ tumors in locations other than GIT, GUT, and CNS and who had recently had surgeries but were significantly less frequently isolated from patients who had hematological malignancies and who were exposed to chemotherapy (OR: 3.657, 95% CI: 1.047 to 12.773, *P* = 0.033; OR: 5.625, 95% CI: 1.624 to 19.478, *P* = 0.003; OR: 0.107, 95% CI: 0.013 to 0.856, *P* = 0.013; OR: 0.234, 95% CI: 0.061 to 0.894, *P* = 0.024, respectively). The former species were significantly more commonly isolated from SSIs (OR: 4.056, 95% CI: 1.262 to 13.037, *P* = 0.013) and less from bloodstream infections (BSIs), with a *p*-value of 0.011.

**Fig. 2:**
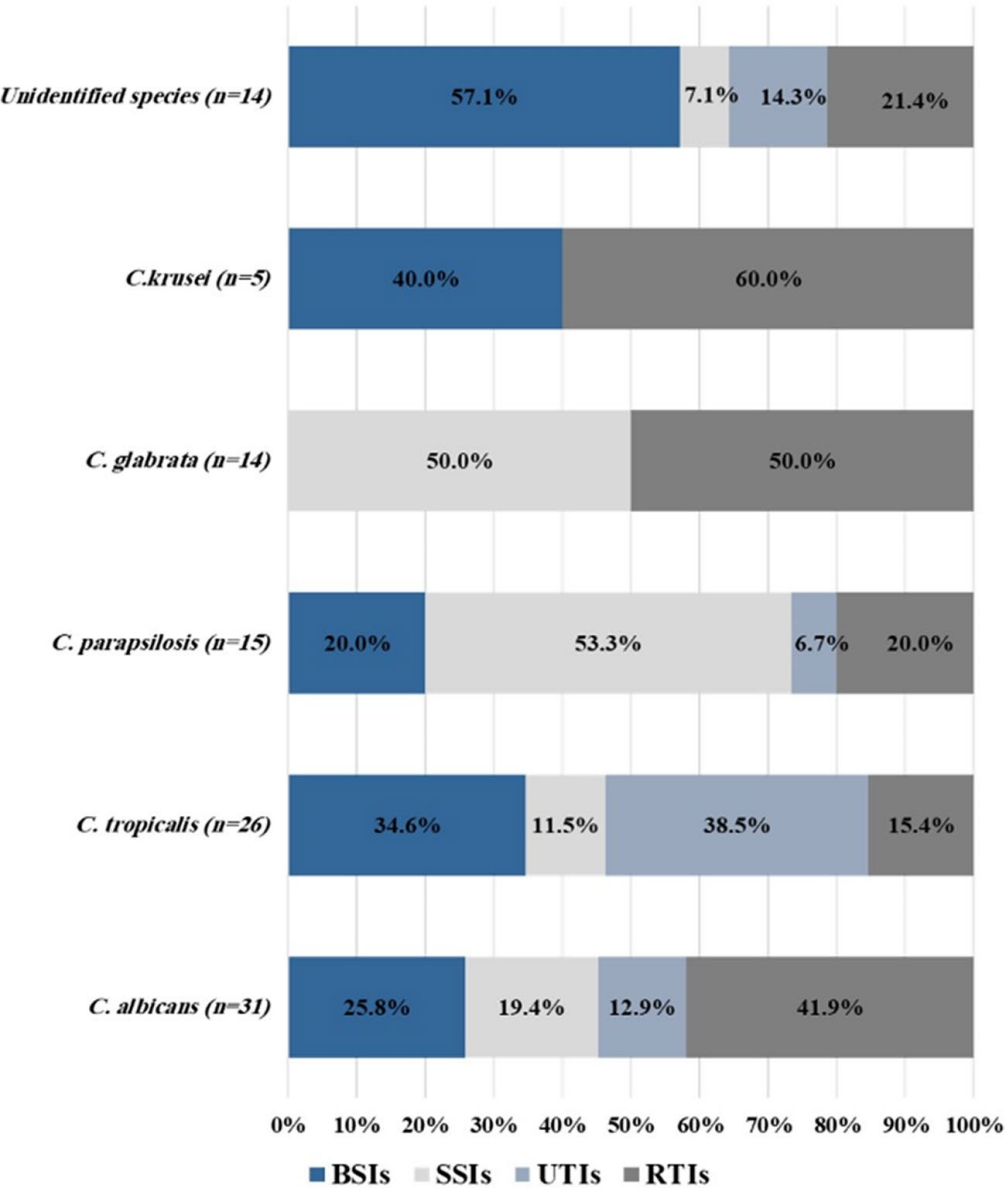
Clinical sites of *Candida* infections in 105 cancer patients. Nearly half of *C. parapsilosis* and *C. glabrata* species infections were SSIs, while 57.1% of patients infected by unidentified *Candida* species had candidemia. BSIs: bloodstream infections, SSIs: surgical site infections, UTIs: urinary tract infections, RTIs: respiratory tract infections

Other *Candida* species infections were significantly less common in patients who had a prolonged hospital stay (≥ seven days) (OR: 0.168, 95% CI: 0.057 to 0.496, *P* = 0.001). That *C. krusei* and unidentified *Candida* species were significantly detected at a higher rate in BSIs and a lower rate in SSIs (OR: 3.667, 95% CI: 1.309 to 10.272, *P* = 0.010; OR: 0.144, 95% CI: 0.018 to 1.135, *P* = 0.036, respectively). Moreover, Fig. 2 illustrates the distribution of clinical sites of infection caused by different *Candida* species among cancer patients. The urinary tract was the most common infection site caused by *C. tropicalis* species (38.5%). However, nearly half of the *C. parapsilosis* and *C. glabrata* species infections were SSIs (53.3% and 50%, respectively). Interestingly, BSI was the most frequent infection produced by unidentified *Candida* species (57.1%, *p* = 0.011).

### Antifungal therapy

Forty-seven patients (44.8%) received antifungal therapy after documented proof of infection. Triazoles (fluconazole or voriconazole) were the most frequently used antifungals (21/47, 44.7%), followed by echinocandins (caspofungin or micafungin) and amphotericin B (*n* = 7, 14.9%, and *n* = 6, 12.8%, respectively). Some patients got more than one antifungal treatment (*n* = 13, 27.7%). All 13 combination antifungal treatments include at least fluconazole or voriconazole. Most of the 58 patients who didn’t receive antifungal treatment (79.3%) had solid organ malignancies (OR: 5.175, 95% CI: 2.192 to 12.218, *P* < 0.001).

### Outcome

**Fig. 3:**
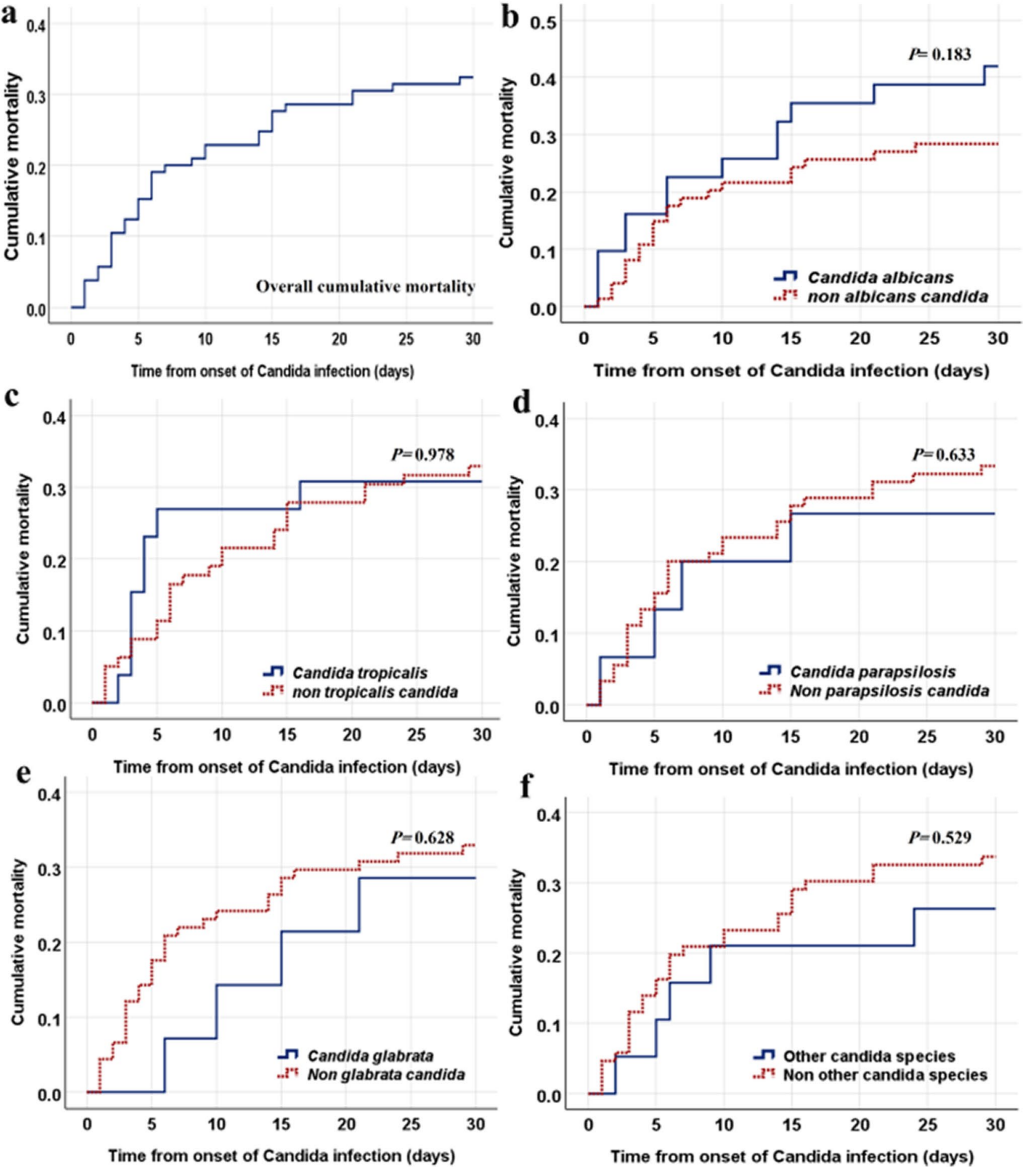
The thirty-day cumulative mortality curves of 105 cancer patients infected with different *Candida* species. The overall 30-day post-infection cumulative mortality curve is shown in (**a**). The cumulative mortality curves for *C. albicans*, *C. tropicalis*, *C. parapsilosis*, *C. glabrata*, and other *Candida* species are also presented in (**b**, **c**, **d**, **e**, and **f**, respectively). Other species include *C. krusei* (*n* = 5) and unidentified *Candida* species (*n* = 14)

The overall thirty-day post-infection cumulative mortality rate was 32.4% (*n* = 34). Figure 3 clarifies the 30-day cumulative mortality of patients infected with different *Candida* species. No significant differences in crude mortality rates were observed among the species groups. Furthermore, univariate regression analysis for associated risk factors for 30-day mortality in *Candida*-infected cancer patients is shown in Table 5. ICU admission and multifocal *Candida* infections were associated risk factors for increased 30-day mortality (OR: 7.167, 95% CI: 2.631 to 19.520, *P* < 0.001; OR: 2.824, 95% CI: 1.187 to 6.715, *P* = 0.017, respectively). However, logistic regression analysis revealed that ICU stay was an independent risk factor for 30-day cumulative mortality in infected patients (adjusted OR: 6.172, 95% CI: 2.210 to 17.231, *P* = 0.001) (Table 6). Thirty-day mortality rates did not differ statistically according to the clinical site of infection; however, the highest rate was seen in patients with candidemia (32.4%, *p* = 0.553).

**Table 5:** Univariate analysis of risk factors for 30-day mortality in 105 *Candida*-infected cancer patients

Risk factors (no patients)	No. of cases (%)		Odds ratio	95% CI	*P*-value
	Death (*n* = 34)	Survival (*n* = 71)			
Age					
< 18 years (*n* = 21)	8 (23.5)	13 (18.3)	1.373	0.508–3.712	0.532
18–50 years (*n* = 38)	8 (23.5)	30 (42.3)	0.421	0.167–1.057	0.062
> 50 years (*n* = 46)	18 (52.9)	28 (39.4)	1.728	0.757–3.941	0.192
Male sex (*n* = 59)	19 (55.9)	40 (56.3)	0.982	0.431–2.237	0.965
Type of cancer					
Hematological malignancies (*n* = 39)	9 (26.5)	30 (42.3)	0.492	0.201–1.205	0.117
Gastrointestinal tract cancer (*n* = 28)	12 (35.3)	16 (22.5)	1.875	0.765–4.598	0.167
Genitourinary tract cancer (*n* = 16)	3 (8.8)	13 (18.3)	0.432	0.114–1.631	0.206
Central nervous system cancer (*n* = 5)	3 (8.8)	2 (2.8)	3.339	0.531–20.995	0.326
Solid cancers in other locations (*n* = 17)	7 (20.6)	10 (14.1)	1.581	0.544–4.596	0.397
Inpatients (*n* = 102)	34 (100.0)	68 (95.8)	NC	NC	0.549
** ≥ **7 days hospital stay (*n* = 69)	20 (58.8)	49 (69.0)	0.641	0.275–1.498	0.303
ICU admission (*n* = 56)	28 (82.4)	28 (39.4)	**7.167**	**2.631–19.520**	** < 0.001**
Recent surgery (≤ 30 days) (*n* = 38)	12 (35.3)	26 (36.6)	0.944	0.402–2.216	0.895
Receiving chemotherapy (*n* = 52)	16 (47.1)	36 (50.7)	0.864	0.381–1.959	0.727
Prior antibiotic use (*n* = 101)	33 (97.1)	68 (95.8)	1.456	0.146–14.537	1.000
No antifungal therapy (*n* = 58)	19 (55.9)	39 (54.9)	1.039	0.457–2.366	0.927
Presence of neutropenia (*n* = 35)	10 (29.4)	25 (35.2)	0.767	0.317–1.856	0.555
Presence of lymphopenia (*n* = 45)	16 (47.1)	29 (40.8)	1.287	0.565–2.932	0.547
*Candida* species					
*C. albicans* (*n* = 31)	13 (38.2)	18 (25.4)	1.823	0.760–4.369	0.176
*C. tropicalis* (*n* = 26)	8 (23.5)	18 (25.4)	0.906	0.348–2.357	0.840
*C. parapsilosis* (*n* = 15)	4 (11.8)	11 (15.5)	0.727	0.214–2.477	0.609
*C. glabrata* (*n* = 14)	4 (11.8)	10 (14.1)	0.813	0.236–2.808	0.744
Other *Candida* species^a^ (*n* = 19)	5 (14.7)	14 (19.7)	0.702	0.230–2.140	0.532
Site of infection					
Blood stream infections (*n* = 30)	11 (32.4)	19 (26.8)	1.309	0.537–3.188	0.553
Urinary tract infections (*n* = 17)	6 (17.6)	11 (15.5)	1.169	0.393–3.481	0.779
Surgical site infections (*n* = 25)	7 (20.6)	18 (25.4)	0.763	0.284–2.051	0.592
Respiratory tract infections (*n* = 33)	10 (29.4)	23 (32.4)	0.870	0.357–2.117	0.758
Multifocal*Candida*infections (*n* = 33)	16 (47.1)	17 (23.9)	**2.824**	**1.187–6.715**	**0.017**

*ICU* intensive care unit, *CI* confidence interval, *NC* non-calculable

^a^other species include *C. krusei* (*n* = 5) and unidentified *Candida* species (*n* = 14). Statistically significant *P*-values are in boldface

**Table 6:** Binary logistic regression analysis of risk factors for 30-day mortality in *Candida*-infected cancer patients

Risk factors	Adjusted		*P*-value
	odds ratio	95% CI	
Age			
18–50 years	0.524	0.191–1.436	0.209
ICU admission	**6.172**	**2.210–17.231**	**0.001**
Multifocal*Candida*infections	2.531	0.968–6.617	0.058

Statistically significant *P*-values are in boldface. *CI* confidence interval, *ICU* intensive care unit

## Discussion

Invasive *Candida* infections have become a public health concern, highly connected to immunocompromised states such as cancer patients. Furthermore, the COVID-19 pandemic has been implicated in a rise in fungal infections [13]. Additionally, there has been a remarkable increase in morbidity and mortality associated with *Candida* infections and decreased susceptibility to commonly used antifungal agents in recent years. This situation necessitates understanding local trends and species distribution among cancer patients with hematological and solid organ malignancies to properly improve the management of those high-risk patients [6].

The changing pattern of *Candida* infections in high-risk cancer patients, manifested by increasing NAC species, is due to previous exposure to antifungals, broad-spectrum antibiotics, chemotherapy, invasive devices, surgeries, prolonged hospital stay, ICU admissions, and the presence of neutropenia [14], in addition to immunocompromised conditions, comorbidities, and the severity of the underlying malignancy in such patients [15]. Furthermore, multidrug-resistant species like *C.* *auris* are becoming more prevalent [13]. This current epidemiology necessitates improving the diagnostic skills of microbiology laboratories. Rapid species identification is necessary because of the increased mortality rates associated with these infections, particularly in high-risk cancer patients [16].

Local trends of *Candida* species are crucial since the variable distribution is observed between geographical regions and is influenced by underlying conditions and risk factors [7]. *Candida* pneumonia diagnosis is challenging because the definitive diagnosis is based on histologic evidence of yeast and inflammatory cells in lung tissue and the failure of less invasive methods to confirm infection. Thus, antimycotic therapy should be reserved for immunocompromised patients with evidence of pneumonia, alongside physicians' clinical judgment [17]. Furthermore, some studies stated that *Candida* detection in the respiratory tract of critically ill patients increased morbidity and mortality, and even if it is thought to be colonization, it may contribute to poor outcomes [18]. Therefore, we included *Candida* isolated from respiratory specimens, particularly considering the immunocompromised state of our patients. In the current study, NAC accounted for 60.6% of the *Candida* identified from thirty-three respiratory samples of cancer patients.

Previous studies from various parts of the world revealed a predominance of NAC species, with varying trends and *Candida* species rankings. In the present study, NAC species accounted for 70.5% of isolates cultured from different body sites. *C. tropicalis* was the predominant cause of NAC infections, followed by *C. parapsilosis*, *C. glabrata*, and *C. krusei* (24.8%, 14.3%, 13.3%, and 4.8%). Our results were comparable to those of the Assiut University Hospitals in Egypt study. In that study, 75% of isolated species were NAC species, with *C. tropicalis* being the most prevalent species (46.5%) cultured from different specimens of ICU patients [19].

Another surveillance study was done in 21 hospitals in seven Latin American countries, where NAC species accounted for 62.4% of candidemia infections. *C. parapsilosis* was the most frequent NAC species isolated (26.5%) [20]. Similarly, a surveillance study in the United States from 2009 to 2017 demonstrated that NAC species caused 52% of IC infections, and *C. glabrata* was the most common one responsible for these infections [21]. On the other hand, a study by Lindberg et al. [22] showed a lower rate of NAC species (35% of all isolates) causing candidemia in the Swedish University Hospital. *C. glabrata* was the most identified NAC species, accounting for 19% of all isolates.

Many studies have reported that the main five *Candida* species we tested (*C.* *albicans*, *C.* *tropicalis*, *C.* *glabrata*, *C.* *parapsilosis*, and *C.* *krusei*) are responsible for over 90% of *Candida* infections [6]. In the current study, 13.3% of *Candida* isolates were not identified by sn-PCR, indicating an increase in infections caused by uncommon *Candida* species among cancer patients. This percentage was higher than in previous surveillance studies conducted in other centers. In a multi-center study involving five years of surveillance of IC in China, 8829 *Candida* isolates from 65 tertiary hospitals were collected. Although 32 *Candida* species were identified using matrix-assisted laser desorption ionization time-of-flight mass spectrometry (MALDI-TOF MS) combined with Vitek MS and DNA sequencing, uncommon *Candida* species contributed 6% (5.7%) of the total isolates [23]. Similarly, Ying et al. [24] revealed rare species accounting for 1.7% (*n* = 53) of the total isolates recovered from various clinical specimens. These isolates were identified by Vitek 2 system.

However, in another study, these rare *Candida* species were detected at a higher rate (10.2%) and identified using PCR-restriction fragment length polymorphism (PCR–RFLP), duplex-PCR, and multiplex-PCR techniques [14]. Interestingly, uncommon species accounted for 21.1% of candidemia in patients with acute leukemia in a cancer center in the USA [25]. Moreover, 31.9% of isolates causing candidemia in patients admitted to the ICU at a tertiary care center in India were uncommon *Candida* species. PCR–RFLP could not identify 32 isolates (26.9% of a total of 119 isolates) but was then identified by MALDI-TOF MS [26]. These findings emphasize the increased rates of NAC species, notably rare species, among high-risk patients. Thus, accurate *Candida* species identification is crucial, particularly for those causing invasive fungal infections (IFIs).

Early antifungal treatment has a significant impact on the patient’s outcome. Thus, a more rapid and accurate diagnosis is crucial [13, 27]. Molecular techniques are more precise, faster, and less liable to growth condition variations and phenotypic changes. Multiplex PCR, PCR–RFLP, and sequencing of specific genome regions are some of the best-known molecular approaches [28]. A broader primer library should be used as *Candida* distribution expands beyond the five major *Candida* species previously known to cause invasive *Candida* infections in high-risk patients [29]. Based on our findings, 13.3% of isolates were not identified using multiplex sn-PCR with the primers of the five common *Candida* species, despite the significant detection of these unidentified species in invasive BSIs (57.1%, *p* = 0.011). In addition, our patients had a high 30-day crude mortality rate of 32.4%. So, probably Vitek, MALDI-TOF, or combined methodologies with a broader identification spectrum are extensively recommended.

Chromagar is an inexpensive and easy method for primarily identifying common *Candida* species, particularly in areas with limited resources. In addition, this medium allows for rapid IC diagnosis and epidemiological surveillance in high-risk units; however, it is still insensitive and unable to identify all species [16]. Moreover, chromagar does not recognize the emergent yeast *C. auris* [13]. Despite the high degree of concordance between chromagar and sn-PCR (80.7%, *p* < 0.001), a discrepancy between the two methods was observed in 13.3% of our isolates. Furthermore, 34.3% of isolates could not be identified to the species level by chromagar since it fails to correctly identify all but three species: *C. albicans*, *C. tropicali*s, and *C. krusei.* In a study conducted in Japan, chromagar could not determine the species of more than half of the *Candida* isolates (66.7%) cultured from various specimens [28]. Thus, the chromogenic medium is unreliable compared to molecular methods because of the inaccurate identification of many *Candida* species [29].

In this study, *Candida* infections were more prevalent in the over-50 age group, with a median age of 50 years among all infected cancer patients. This was in agreement with previous studies [6, 22, 27]. Most *Candida* isolates in our study were from hospitalized patients (97.1%). Other risk factors for *Candida* infection observed in our patients included a prolonged duration of hospitalization of ≥ 7 days (65.7%), an ICU stay (53.3%), previous antibiotic exposure (96.2%), chemotherapy (49.5%), and recent surgeries (36.2%). Similarly, it was reported that the age of less than one or more than sixty-five years, a central venous catheter (CVC), surgical procedures, exposure to chemotherapy, and prior antibiotic uptake are all main risk factors for invasive *Candida* infections [1, 30]. Furthermore, invasive *Candida* infections were significantly higher in patients with prolonged hospital stays and genitourinary tract cancer (*p* = 0.005 and *p* = 0.049, respectively). Similarly, Xia et al. [31] found that increased hospitalization duration was significantly associated with invasive *Candida* infections (*p* = 0.037). Additionally, invasive infections were significantly more often detected in patients with cancer as an underlying comorbidity (*p* = 0.006) [31].

In our study, the incidence of *C. glabrata* and *C. parapsilosis* infections was significantly higher in patients who had recent surgeries, and they were significantly revealed much more frequently from SSIs. Infections with *C. tropicalis* species were significantly associated with hematological malignancies and were more commonly detected in UTIs. Negri et al. [32] also stated the link of *C. tropicalis* with UTIs and hematological malignancies. Furthermore, Lortholary et al. [33] and Wu et al. [2] also reported a significant association between *C. tropicalis* and hematological malignancies. Interestingly, unidentified *Candida* species were significantly more frequently isolated from BSIs (*p* = 0.011), emphasizing the importance of accurate *Candida* species identification.

In our study, the thirty-day crude mortality rate of infected cancer patients was 32.4%, with candidemia patients having the highest mortality rate of 32.4%. Reports from other studies showed nearly the same mortality rates of 30% to 40%. At 28 days, mortality was 26.3% among candidemia-infected pediatric cancer patients [1]. Similarly, a prospective multicenter study of invasive candidiasis found a 30-day mortality rate of 38.8% among ICU surgical patients [34]. Another study in China on cancer patients with candidemia showed a 30.0% mortality rate at 30 days. This rate was significantly higher than and roughly twice as high as bacterial BSIs (*p* = 0.006) [35]. In agreement with previous studies [30, 33], we revealed that ICU admission was an independent risk factor for increased 30-day mortality (*p* = 0.001).

## Conclusion

NAC species infections predominate, with increasing rates of rare *Candida* species other than the five common species (*C. tropicalis, C. albicans*, *C. glabrata*, *C. krusei, and C. parapsilosis*), particularly in critically ill patients. This epidemiology necessitates rapid, accurate species identification and an understanding of local trends, distribution, and risk factors associated with these pathogens for optimal management of cancer patients. Multiplex sn-PCR is one of the best-known molecular techniques that could be used for species identification, while chromagar is an unreliable method compared to PCR. Other techniques with an extended identification spectrum, such as MALDI-TOF or combined methods, are still highly recommended to deal with the increasing rates of uncommon species and their significant involvement in invasive infections. Patients with hematological and solid organ tumors were at risk of developing IC, with the main risk factors being previous exposure to antibiotics, prolonged hospitalization, ICU, recent surgeries, and chemotherapy. Underlying cancers, chemotherapy, and recent surgeries were all risk factors significantly linked to infection with a given species. Moreover, ICU and multifocal *Candida* infections were significant risk factors for higher mortality rates, with ICU being independently associated with 30-day post-infection mortality. We provide a thorough overview of *Candida* infections at various body sites. Our institute treats cancer patients from all over the country. So, we suggest our results represent this high-risk group of patients in Egypt.

## Data Availability

The corresponding author will provide the required data upon reasonable request.
